# High Morbidity during Treatment and Residual Pulmonary Disability in Pulmonary Tuberculosis: Under-Recognised Phenomena

**DOI:** 10.1371/journal.pone.0080302

**Published:** 2013-11-29

**Authors:** Anna P. Ralph, Enny Kenangalem, Govert Waramori, Gysje J. Pontororing, Sandjaja, Emiliana Tjitra, Graeme P. Maguire, Paul M. Kelly, Nicholas M. Anstey

**Affiliations:** 1 Global and Tropical Health Division, Menzies School of Health Research and Charles Darwin University, Northern Territory, Australia; 2 Department of Infectious Diseases, Division of Medicine, Royal Darwin Hospital, Northern Territory, Australia; 3 Menzies School of Health Research-National Institute of Health Research and Development Research Program, Timika, Papua Province, Indonesia; 4 District Health Authority, Timika, Papua Province, Indonesia; 5 Public Health & Malaria Control Department, PT Freeport Indonesia, Timika, Papua Province, Indonesia; 6 National Institute of Health Research and Development, Jakarta, Indonesia; 7 Baker IDI Heart and Diabetes Institute, Alice Springs, Northern Territory, Australia; 8 School of Medicine and Dentistry, James Cook University, Cairns, Queensland, Australia; 9 Population Health Division, ACT Government Health Directorate, Canberra, Australian Capital Territory, Australia; 10 Australian National University Medical School, Canberra, Australian Capital Territory, Australia; Institute of Infectious Diseases and Molecular Medicine, South Africa

## Abstract

**Background:**

In pulmonary tuberculosis (PTB), morbidity during treatment and residual pulmonary disability can be under-estimated.

**Methods:**

Among adults with smear-positive PTB at an outpatient clinic in Papua, Indonesia, we assessed morbidity at baseline and during treatment, and 6-month residual disability, by measuring functional capacity (six-minute walk test [6MWT] and pulmonary function), quality of life (St George’s Respiratory Questionnaire [SGRQ]) and Adverse Events ([AE]: new symptoms not present at outset). Results were compared with findings in locally-recruited volunteers.

**Results:**

200 PTB patients and 40 volunteers were enrolled. 6WMT was 497m (interquartile range 460-529) in controls versus 408m (IQR 346-450) in PTB patients at baseline (p<0.0001) and 470m (IQR 418-515) in PTB patients after 6 months (p=0.02 versus controls). SGRQ total score was 0 units (IQR 0-2.9) in controls, versus 36.9 (27.4-52.8) in PTB patients at baseline (p<0.0001) and 4.3 (1.7-8.8) by 6 months (p<0.0001). Mean percentage of predicted FEV_1_ was 92% (standard deviation 19.9) in controls, versus 63% (19.4) in PTB patients at baseline (p<0.0001) and 71% (17.5) by 6 months (p<0.0001). After 6 months, 27% of TB patients still had at least moderate-severe pulmonary function impairment, and 57% still had respiratory symptoms, despite most achieving ‘successful’ treatment outcomes, and reporting good quality of life. More-advanced disease at baseline (longer illness duration, worse baseline X-ray) and HIV positivity predicted residual disability. AE at any time during treatment were common: itch 59%, arthralgia 58%, headache 40%, nausea 33%, vomiting 16%.

**Conclusion:**

We found high 6-month residual pulmonary disability and high AE rates. Although PTB treatment is highly successful, the extent of morbidity during treatment and residual impairment could be overlooked if not specifically sought. Calculations of PTB-related burden of disease should acknowledge that TB-related morbidity does not stop at 6 months. Early case detection and treatment are key in minimising residual impairment.

## Introduction

The morbidity experienced by people with pulmonary tuberculosis (PTB) can be evaluated using measures such as pulmonary function testing, quality of life questionnaires and assessment of treatment complications. Quality-adjusted life year calculations (QALY), which provide a measure of the burden of tuberculosis (TB)-related disease, could underestimate true PTB morbidity if they assume a short disease duration [1,2], or do not incorporate long-term pulmonary disability resulting from permanent lung damage [3]. Under-estimates in morbidity arising from TB disease or TB medications may also occur in clinical or research settings in which passive reporting of symptoms is relied upon, or in which only serious adverse effects (requiring medication cessation [4-6]) are captured. The requirement for strict adverse event reporting in clinical trials provides an important opportunity to gather detailed information about symptoms experienced during TB treatment. 

TB is a well-recognised independent risk factor for chronic obstructive pulmonary disease (COPD) [7-11]. Both early, reversible lung impairment in pulmonary TB [8,12] and longer-term residual impairment after TB [8,10] are evaluable using spirometric measures such as percentage of predicted forced expiratory volume in 1 second (FEV_1_). We have previously reported high rates of residual pulmonary disability in PTB patients in eastern Indonesia [8]. At treatment completion, a quarter of successfully-treated patients still had significant lung function impairment (FEV_1_<60% predicted, using local healthy volunteers as the reference population for calculation of percentage of predicted FEV_1_ [13]). Late residual pulmonary disability has been reported by Pasipanodya et al, showing that people with previous active TB were 5.4 times more likely to have abnormal pulmonary function than people without previous active tuberculosis [10].

Quality of life questionnaires can also reveal a fuller scope of PTB morbidity than is obtainable from standard severity measures such as sputum microscopy grade or radiological disease extent. The St George’s Respiratory Questionnaire (SGRQ) has been shown to be an effective tool for measuring PTB morbidity [14]. The SGRQ is a respiratory health-related quality of life (QoL) instrument formulated for use in COPD [15,16], but validated in TB [8,14] and other respiratory diseases. It has been translated into many languages including Indonesian [16], with minor modifications for local suitability.

This study was performed within the context of a clinical trial of nutritional interventions, where detailed information on symptoms, intercurrent illness and disease burden was collected. In order to gain an holistic understanding of the scope of morbidity during TB treatment in an outpatient, resource-limited, high TB-burden setting, our objectives in this paper were to investigate morbidity at baseline and during follow up (symptoms, Adverse Events [AE], functional capacity and QoL), and residual disability at 6 months, experienced by adults with PTB. We furthermore sought to identify predictors of residual disability, and compare functional and QoL measures in PTB with reference ranges established from locally-recruited healthy controls. 

## Methods

This study was performed within a clinical trial of adjunctive nutritional supplements (L-arginine and vitamin D) for PTB (clinicaltrials.gov/NCT00677339). In this study, the interventions did not significantly affect outcome measures including AE [17]. Eligible sequential study participants with PTB who had no previous history of treatment were enrolled at the tuberculosis clinic in Timika, Indonesia. Inclusion criteria included age ≥15 years, sputum smear-positive for acid fast bacilli (AFB), and provision of written informed consent. The standard 6-month tuberculosis treatment regimen [18] was administered in a directly observed fashion. 

Local healthy controls were eligible if they were aged ≥18 years, gave written informed consent, and had no co-morbidities. Volunteers were approached via community contacts and social groups from among friends and relatives of staff members or patients (after undergoing TB contact tracing and if found not to have TB). Volunteers were eligible as healthy controls if they passed a symptom screen and assessment of pulse and blood pressure. They were ineligible if they had ‘any current sickness’, cough, angina, fever or any febrile illness within the last week, pulse >120/min, systolic blood pressure >180mmHg or diastolic blood pressure >100 mmHg. The latter exclusions were to ensure safety for physical exertion to perform 6MWT. We did not ask minors aged <18 years to participate, due to the requirement for them to attend the clinic solely for research purposes and to undergo blood tests and other procedures (in contrast to TB patients needing to attend the clinic anyway), especially given potential difficulties in locating a parent/guardian to provide consent.

### Ethics statement

The study was approved by the Human Research Ethics Committees of Menzies School of Health Research, Darwin, Australia and the National Institute for Health Research and Development, Jakarta, Indonesia. Written informed consent was obtained from the participant (and guardian if the participant was aged <18 years) after discussion in Indonesian or a relevant Papuan language aided by pictorial and written information.

### Setting

Timika, in southern Papua Province, Indonesia, population ~200,000, comprises approximately half Indigenous Papuans and half Non-Papuan Indonesians. Papua is relatively disadvantaged within Indonesia, having higher TB (311/100,000 [19]) and HIV rates [20]. Population HIV seroprevalence among Papuans was estimated at 2.4% in 2006 [21]. Indonesian smoking rates have been estimated at 67% of men and 4.5% of women [22]. Traditional Papuans use indoor wood fires for cooking and heating; however, Papuans living in the urban setting of Timika no longer tend to live in traditional huts with internal cooking fires. In a previous study in Timika [8], exposure to indoor smoke was not found to be associated with pulmonary function (unpublished data). 

### Definitions and Procedures

Symptoms were assessed at each visit using a comprehensive checklist. AE were defined, in keeping with Good Clinical Practice (GCP), as any serious or non-serious untoward medical occurrence which had not been noted at baseline, regardless of perceived relatedness to medications or to TB [23]. Such an occurrence reported at least once at any time during TB treatment was documented as an AE. In keeping with national practice, liver function tests were not routinely monitored [24]. All participants were offered HIV testing on venous blood using rapid point-of-care tests. Anaemia was defined as >13.5 g/dL in males, >11.5 g/dL in females. Where malaria co-infection was suspected, diagnosis was by blood film. Sputum stained using the Ziehl-Neelsen method was examined for acid fast bacilli at the Timika field laboratory weekly for 8 weeks then at weeks 20 and 24. Treatment outcome was categorised as successful if the patient successfully completed or was cured (smear-negative in the last month of treatment and on at least one previous occasion) [25]. 

Postero-anterior chest radiographs were scored according to a previously-reported method, the Timika TB x-ray score [26]. The Indonesian Modified SGRQ was used to assess QoL; this has previously been tested in PTB patients in Timika [8]. A score of 0 indicates no lung-related QoL impairment; 100 represents severe impairment. A change of 4 units is considered clinically significant [27]. Pulmonary function (forced vital capacity [FVC] and forced expiratory volume in 1 second [FEV_1_]) was measured using a handheld spirometer (MicroLoop®, MicroMedical, UK), with individual-use filtered one-way mouthpieces (Sure-Gard®). The percentage of predicted FEV_1_ was calculated from previously-established local normal reference ranges [13]. The 6-minute walk test (6MWT) was assessed on an outdoor track according to American Thoracic Society guidelines [28]. A ‘learning effect’ of 5-12% is recognised in serial 6MWT performed approximately 20 minutes apart [29,30], but such an effect is unlikely to be seen when tests were separated by at least 4 weeks, as here, and if present, would bias the interpretation of residual disability results towards the null. Lung function impairment categories were defined as: normal ≥80% predicted FEV_1_; mild lung function impairment 70-79% predicted, moderate 60-69%, Moderate-severe 50-59%, severe 35-49%, very severe <35% [31]. Pulmonary function, 6MWT and the SGRQ were undertaken at baseline then at 0, 4, 8 and 24 weeks and chest radiographs at 0, 8 and 24 weeks. Pulmonary function, 6MWT and SGRQ scores at 6 months were used to assess of the presence of residual disability. 

### Statistical methods

Analyses were undertaken using Stata 12.1, StataCorp, Texas, USA. To calculate adverse event rates, participants who attended less than half of the scheduled follow up appointments (<6) were excluded. Age was grouped as ≤35 or >35 years where required, since age >35 is associated with higher AE risks in active [4] or latent [32,33] TB treatment. To calculate percentage changes over time in serial measures, participants for whom results were unavailable at baseline or 6 months were excluded. Proportions were compared using Chi-squared test, or Fisher’s exact test where necessary. For testing associations between continuous and categorical variables, Student’s 2-sample T test or Wilcoxon rank sum test were used depending on the distribution. For testing associations between two continuous variables, univariable and multivariable regression models were used if variables were normally distributed, or Spearman’s Rho and correlation matrices, with Bonferroni correction for multiple comparisons, if non-normally distributed and not amendable to transformation. Multivariable models were constructed using a backwards stepwise approach. Variables included in the initial model were those significantly associated in univariable analysis with the independent variable, or plausibly related to the independent variable. All tests were two-sided with a P value <0.05 considered to be statistically significant.

## Results

Study participants and healthy volunteers were enrolled from June 2008 to February 2010, as described elsewhere [17,34,35].. Data from all 200 participants included in the overall trial are included here [36]. Compared with TB patients, volunteers were adequately matched according to sex, age, ethnicity and height, but had higher body weight, as expected (Table 1). 

**Table 1:** Participant characteristics.

	**TB patient**	**Healthy volunteer**	**P value**
**n**	200	40	
**Age in years: median (IQR**)	28 (23-36.5)	26.5 (23.5-33)	0.7
**Female: number (%)**	69 (34.5)	9 (22.5)	0.1
**Papuan: number (%**)	89 (44.5)	20 (50.0)	0.5
Female: number (%)	29 (32.6)	6 (30.0)	1.0
**Current or ex-smoker: number (%)**	109 (55%)	19 (48%)	0.4
**Height in metres: mean (SE)**	1.61 (0.01)	1.58 (0.01)	0.08
**Weight in kg: mean (SE)**	62.5 (1.63)	48.5 (0.54)	<0.0001
Successful TB treatment outcome (cured/completed)*	146/186 (79%)	-	-
Multidrug-resistant TB*	2/149 (1.3)	-	-

^*^ Outcome category and culture and susceptibility results were unavailable in some participants

### Symptoms, functional capacity and quality of life among TB patients

**Figure 1:**
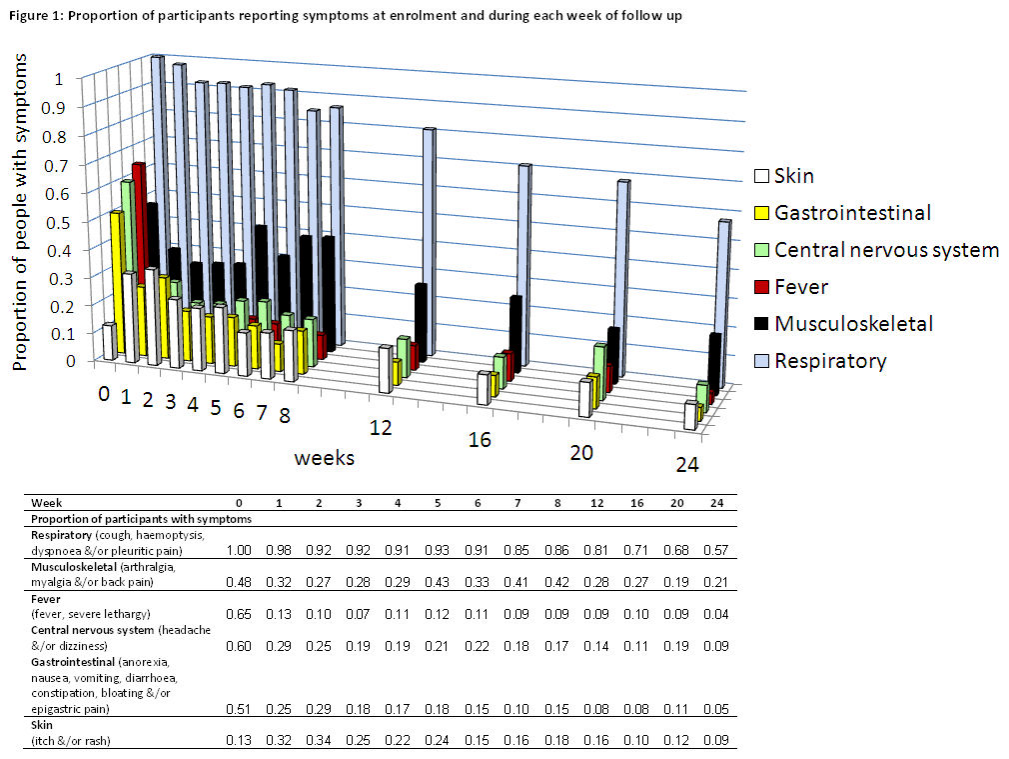
Proportion of participants reporting symptoms at enrolment and during each week of follow up.

**Figure 2:**
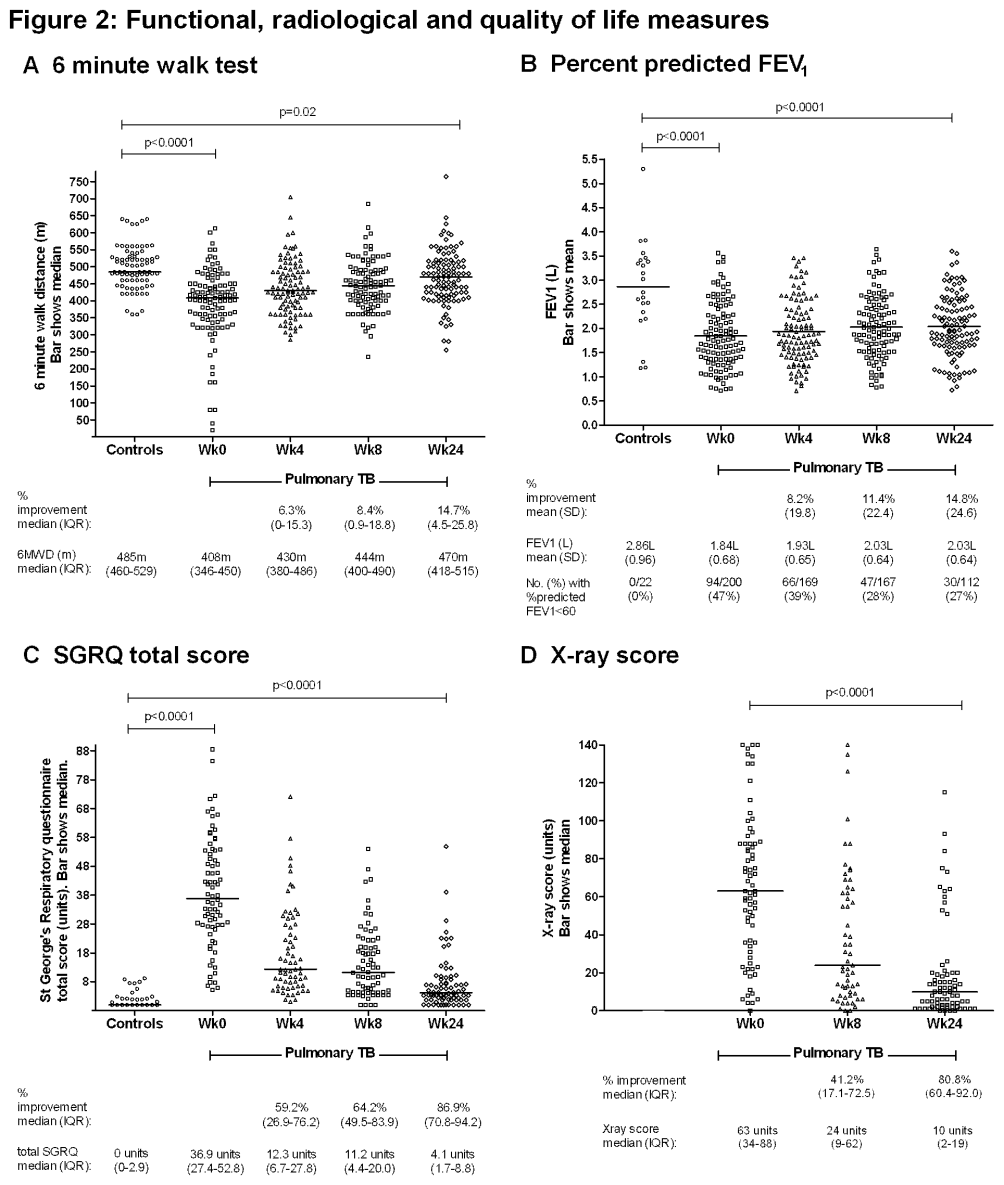
Functional, radiological and quality of life measures. *Data shown only for participants who had measures performed both at enrolment of 6 months (numbers shown in brackets). A 6 minute walk test (n=107) B Percentage of predicted forced expiratory volume in 1 second (n=112) C St George’s respiratory questionnaire total score (n=76) D X-ray score (n=73)

PTB patients were most symptomatic and had greatest functional and QoL impairment at baseline, as expected; all measures significantly improved with TB treatment (Figures 1 and 2). Almost 80% of people had a successful treatment outcome (Table 1). However, no measures other than sputum microbiology showed complete normalisation by 6 months. Despite rapid resolution of fever, lethargy, headache and dizziness, other symptoms, while mostly decreasing over time, remained well-represented, with over half the participants still having respiratory symptoms at treatment completion (Figure 1). Musculoskeletal symptoms (chiefly arthralgia) eased initially, then increased up until week 8 before decreasing again; this was frequently attributed by clinical staff to pyrazinamide. Baseline symptoms were un-associated with age, sex, ethnicity or HIV status (data not shown). 

Functional capacity (6MWT, percent predicted FEV_1_), QoL (SGRQ) and chest X-ray results are shown in Figure 2. Those who were most unwell walked very short distances, creating a skewed distribution in 6MWT data (Figure 2A). All participants included in this study were ambulant as they were all manageable in an outpatient setting (needing to be able to walk into the clinic), but the most unwell had very limited exercise tolerance, being only able to walk minimal distances. Four people walked for only 55 metres or less, stopping well before 6 minutes, and another 9 people could only walk for 200m or less. 6MWT, %predicted FEV_1_ and SGRQ were each still significantly worse among PTB patients at treatment completion than in the healthy controls. Only small percentage improvements were achieved in %predicted FEV_1_ and 6MWT (14.8% and 14.7% respectively) by TB patients over the 6 months; by 6 months, 27% of TB patients still had at least moderate-severe pulmonary function impairment (Figure 2B). More substantial improvements occurred in serial SGRQ and Timika TB x-ray scores (Figures 2C and D). The median FEV1/FVC ratio was >0.70 (not suggestive of an obstructive pattern [37]) and did not differ between TB patients at baseline (0.87, range 0.51-1.00) and controls (0.86, range 0.69-0.99). Nine TB patients (4.5%) and 1 control (4.5%) had FEV1/FVC ratio <0.70.

### Predictors of residual disability

In univariable analyses, HIV+ status was significantly associated with worse QoL (SGRQ) and shorter 6MWT at 6 months; the length of patient-reported diagnostic delay prior to treatment commencement (‘illness duration’) was significantly associated with residual lung function impairment, x-ray score and weight at 6 months; baseline Timika TB x-ray score was significantly associated with final x-ray score and lung function impairment at 6 months (Table 2). No other significant associations between baseline demographic/clinical variables and 6-month outcomes were identified. In multivariable models, HIV status was no longer associated with 6WMT, and the associations between illness duration and 6-month outcomes were attenuated (Table 2). 

**Table 2:** Associations between baseline measures and residual disability at 6 months.

**Baseline variable**	**Six-month outcome**	**p value**
HIV status*	**Six-month St George’s Respiratory Questionnaire total score: median (IQR)**	
	HIV- : 3.25 units (1.72-7.18)	0.02
	HIV+ : 15.8 units (7.01-29.2)	
	**Six-month Six-minute walk test: median (IQR)**	
	HIV- : 480m (425-520)	0.6
	HIV+ : 440m (420-475)	
**Illness duration (diagnostic delay**)** prior to commencing treatment** †	**Six-month Weight: mean (SD)**	
	Illness duration ≤3 months‡: 54.5kg (8.4)	0.04
	Illness duration >3 months: 51.1kg (7.3)	
	**Six-month X-ray score: median (IQR)**	
	Illness duration ≤3 months: 6 units (2-14)	0.05
	Illness duration >3 months: 19 units (6-65)	
	**Six-month %Predicted FEV_1_: mean (SD)**	
	Illness duration ≤3 months: 75.4% (15.8)	0.06
	Illness duration >3 months: 62.9% (18.3)	
**X-ray score at treatment commencement** †	**Six-month X-ray score: median (IQR**)	
	X-ray score ≤70‡: 5 units (1-11)	<0.0001
	X-ray score >70: 10.5 units (2-19)	
	**Six-month %Predicted FEV_1_: mean (SD)**	
	X-ray score ≤70: 78.2% (13.3)	<0.0001
	X-ray score >70: 64.2% (19.1)	

^*^ p values calculated using multivariable logistic regression

^†^ p values calculated using pairwise correlation between continuous variables with Bonferroni correction for multiple comparisons

^‡^ continuous explanatory variables have been dichotomised to illustrate differences.

### Establishment of local normal reference ranges

**Figure 3:**
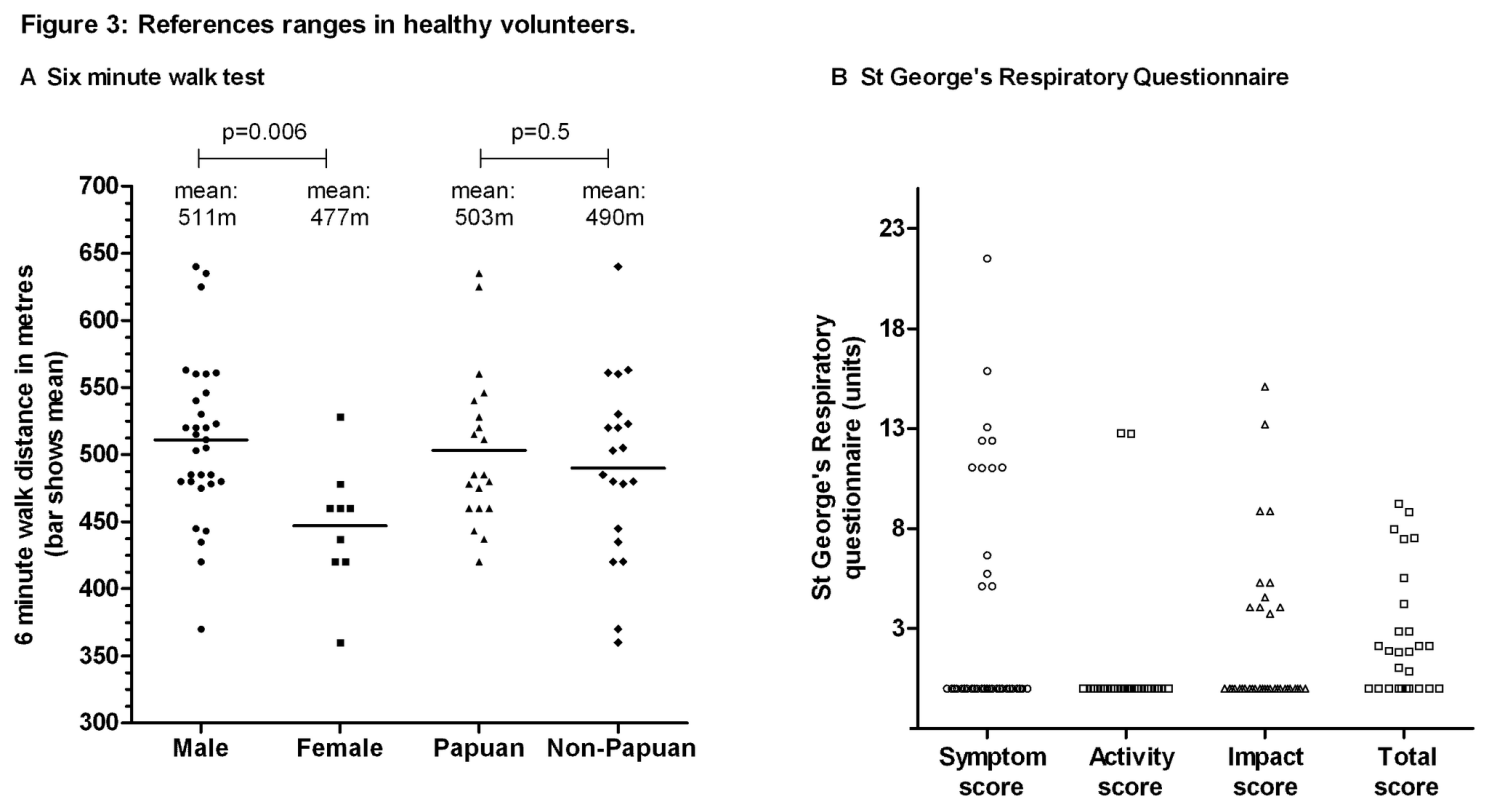
References ranges in healthy volunteers. A Six minute walk test. B St George’s Respiratory Questionnaire.

The healthy volunteers had a mean 6MWT distance of 497±63m (range 360-640m). Males walked further than females; no significant difference in distance was observed between ethnic groups (Table 3, Figure 3A). Modified SGRQ scores (total and individual domains) were obtained in 35 volunteers, the median total score being 0 (range 0-9.23), (Table 3, Figure 3B) with no differences between sex or ethnic groups in total or domain scores. 

**Table 3:** Normal reference ranges for 6 minute walk test and modified (Indonesian) St George’s Respiratory Questionnaire in Timika, Papua, Indonesia*.

	**All**	**Male**	**Female**	**Papuan**	**Non-Papuan**
**St George’s Respiratory Questionnaire (Units**)**: median (range**)					
Symptom score	0 (0-21.51)	0 (0-21.51)	0 (0-12.39)	0 (0- 21.51)	0 (0-13.07)
Activity score	0 (0-12.78)	0 (0-12.74)	0 (0-12.78)	0 (0- 12.78)	0 (0-0)
Impact score	0 (0-15.1)	0 (0-15.1)	0 (0-3.73)	0 (0- 8.87)	0 (0-15.1)
Total score	0 (0-9.23)	0.86 (0-9.23)	0 (0-7.97)	1.88 (0- 7.97)	0 (0-9.23)
**6-minute walk test (m**)**: mean (SD**)†	497 (63)	511 (60)	477 (46)	503 (58)	490 (69)

^*^ 6MWD and total SGRQ score summary statistics in these healthy controls, without sex or ethnic group breakdown, have been previously cited [34]

^†^ 6-minute walk test male vs female p=0.006. No other significant differences in SGRQ or 6MWT between sex or ethnic groups.

### Intercurrent illness

Among the 200 study participants with PTB, there were 2237 clinical reviews (on average, 11 appointments per participant). Nineteen participants (13%) were HIV positive (also reported in [20]). One hundred thirty three (55%) had anaemia at baseline, which had decreased to 26% of people by week 24 [35]. Twenty two episodes of malaria were recorded in this region of unstable malaria transmission [38]: 8 *Plasmodium falciparum*, 8 *Plasmodium vivax*, 6 unspecified. These included three Papuan individuals (2 males, 1 female) who each had 2 discreet episodes of fever, with different malarial species reported on blood films (*P. falciparum* then *P.vivax* or vice versa), at intervals of 4, 8 and 13 weeks respectively. An additional 11 people had symptoms consistent with malaria, but negative blood films. 

### Adverse Events

**Figure 4:**
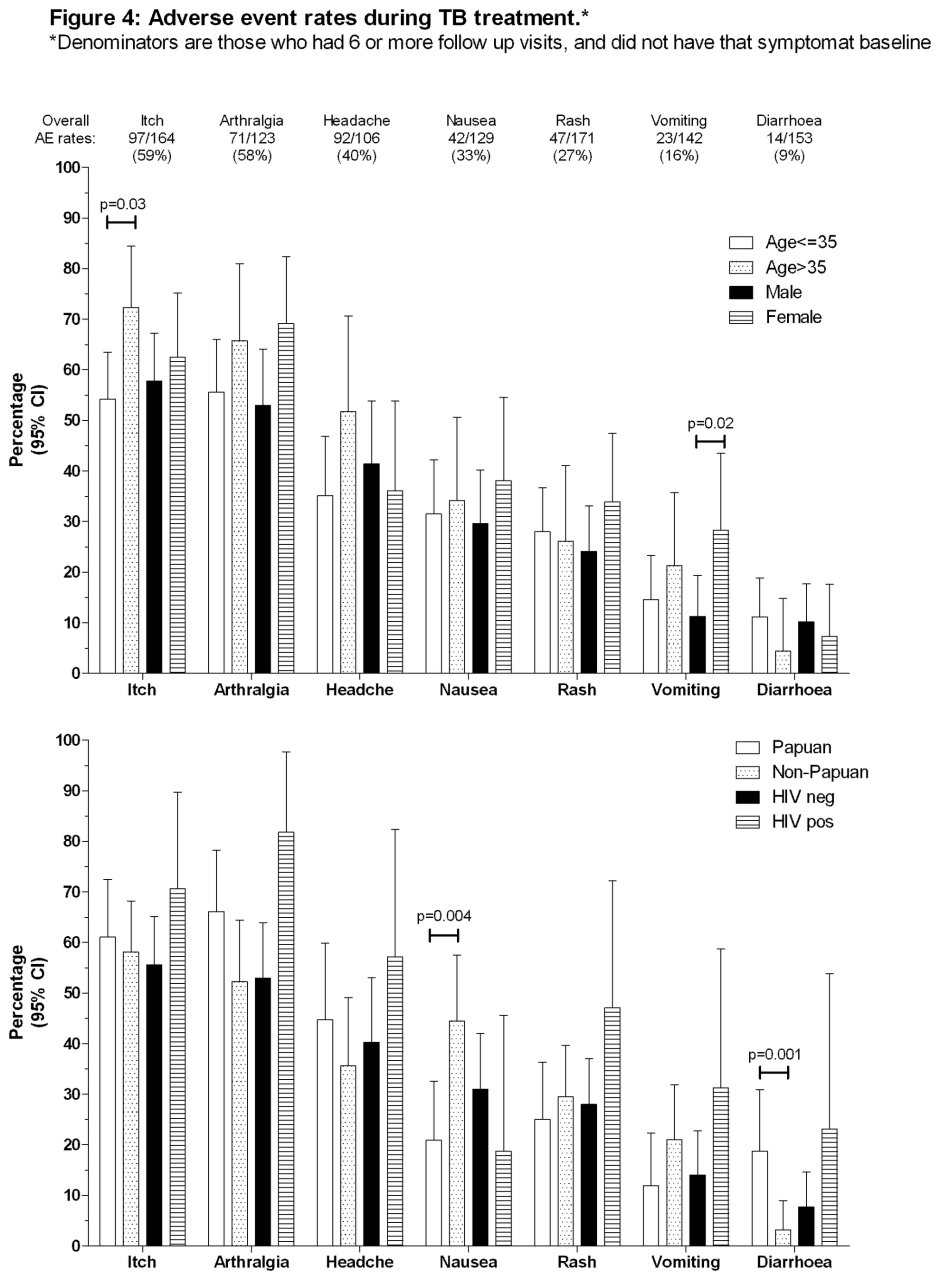
Adverse event rates during TB treatment.

Two participants had TB medications withheld or ceased due to AE; 1 due to rash (attributed to both pyrazinamide and ethambutol), the other due to vomiting (attributed to rifampicin). Excluding participants who attended <6 follow up appointments, the proportions who experienced AE during follow up are shown in Table 4 and Figure 4. Participants who attended <6 follow up appointments did not differ demographically or clinically from those who attended ≥6 appointments (data not shown). Commonest AE were itch and arthrlagia. Few predictors of AE were identified: itch was more common in people aged >35 than those ≤35 years; nausea was more common but diarrhoea less common in people of Non-Papuan than Papuan ethnicity; vomiting was more common in females than males. 

**Table 4:** Adverse event rates in a range of study types and locations.

**Adverse Event**	**Proportion**	**Rate**	**Study participant mean or median age (years)**	**Study type**	**Reference**
**Arthralgia**					
Non-severe	71/123*	58%	28	Prospective observational	This study, Indonesia, 2013
	44/165†	27%	31	Randomised trial	Burman et al, Africa and North America, 2006 [47]
Severe‡	13/519	2%	44	Retrospective observational	Schaberg et al, Germany, 1996 [5]
**Nausea**					
Non-severe	42/129	33%	28	Prospective observational	This study
	15/165	9%	31	Randomised trial	[47]
	1/24	4%	33	Randomised trial in multi-drug resistant TB	Diacon et al, South Africa, 2009 [48]
Severe	5/519	0.9%	44	Retrospective observational	[5]
**Rash**					
Non-severe	47/171	27%	28	Prospective observational	This study
Severe	33/519	6%	44	Retrospective observational	[5]
	18/430	4%	40	Retrospective observational	Yee et al, Canada, 2003 [4]
**Vomiting**					
Non-severe	23/142	16%	28	Prospective observational	This study
	15/165	9%	31	Randomised trial	[47]
	2/24	8%	33	Randomised trial	[48]
**Diarrhoea**					
Non-severe	14/153	9%	28	Prospective observational	This study
	6/165	4%	31	Randomised trial	[47]
	1/24	4%	33	Randomised trial	[48]
**Temporary or permanent cessation of TB medication due to adverse event**	2/200	1%	28	Prospective observational	This study
	35/332	11%	31	Randomised trial	[47]
	0/47	0%	33	Randomised trial	[48]
	121/519	23%	44	Retrospective observational	[5]
	51/1149	4%	36	Retrospective observational	Gulbay et al, Turkey, 2006 [6]

^*^ For the current study, denominators are the subset of the 200 patients who had ≥6 follow up visits, and did not have that symptom at baseline

^†^ For randomised trials, adverse event rates are given for the standard-treatment arm where a novel comparison arm was used

^‡^ Severe = requiring drug cessation

## Discussion

We have shown high morbidity and residual disability amongst ambulatory outpatient PTB patients, in whom treatment outcomes were mostly considered successful, and drug-resistant TB rates were low (Table 1). After 6 months of treatment, the majority of participants had impaired functional or QoL scores compared to the average healthy control, a quarter had at least moderate-to-severe pulmonary function impairment (Figure 2B), and 57% of study participants still had respiratory symptoms (Figure 1). Thus 6-month residual disability, especially that assessed using objective functional measures (6MWT and pulmonary function), is very prevalent in this setting after the standard duration of treatment for PTB. People with advanced disease at baseline (prolonged diagnostic delay or advanced x-ray changes), and those with HIV, were most likely to suffer residual disability (Table 2), emphasising the critical importance of early diagnosis and treatment initiation, and optimised management of HIV co-infection [20]. Our findings are supported by previous investigations calling for recognition in the calculation of TB-related QALYs that TB-related morbidity does not stop after 6 months [3,8]. 

It is possible that at least some pulmonary impairment among TB patients was pre-existing, and that pulmonary disability at 6 months reflected their pre-TB level of function, especially given that smoking and COPD are risk factors for TB. However, based on our findings here, and previous studies [3,10], it is very plausible that the added burden of pulmonary impairment attributable to TB, which was at least partly reversible, contributed to the large disparity between TB patients at treatment completion and controls. Also, the fact remains that at TB treatment completion, patients still have important ongoing health care requirements related to lung disease, be they pre-existing or resulting from the PTB. We did not collect data on exposure to indoor air pollution from cooking fires; however, this would have affected Papuans disproportionally compared with Non-Papuans who do not traditionally use indoor fires, whereas we found no difference in lung function impairment in the ethnic groups.

Adverse event rates in this study are much higher than reported elsewhere, although medication cessation due to AE was uncommon. AE rates vary widely among different studies (Table 4), potentially attributable to differing study designs, AE documentation method, average participant age, and thresholds among staff and patients to tolerate adverse effects before reporting them or withholding medications. Much literature only reports TB drug side-effects serious enough to require drug cessation [4-6,39]. Our high rates of non-serious AE might relate to the particular GCP definition used (see Methods) [23]; that participants were actively questioned about symptoms at each visit; cultural factors among staff and patients regarding symptom documentation; and intercurrent illness. Other researchers use tighter definitions of AE (e.g. events ‘possibly or probably related to treatment’ [40]). Regardless of the aetiology of documented AE in this study, these data offer a real-world perspective on the high burden of illness suffered during a 6-month period by people undergoing TB treatment in a low-resource setting. This is likely to be generalisable to other TB-endemic settings, but may pass unrecognised if only sputum microscopy, chest x-ray and weight are tracked, or if only severe symptoms, or those detected passively, are reported. Awareness of non-serious AE is important regarding their potential impact on adherence, even in directly-observed treatment (DOT) programs, since ‘DOT’ may comprise weekly or, in the continuation phase, monthly, clinic supervision, with opportunities for incomplete adherence between appointments if AE are present. 

Increasing age is recognised to be a major risk factor for serious TB medication side effects (chiefly isoniazid-related hepatotoxicity)[4,32,33,39], and female sex is also identified as an occasional predictor of serious adverse event risk [4,39]. We did not find consistent associations between these or other factors and the occurrence of non-serious AE (Figure 4). 

We established normal reference ranges for 6MWT and SGRQ in this population for the first time. The healthy Timika volunteers walked a substantially shorter mean distance (497m) than healthy people elsewhere (571-659m [29,41,42]). Factors other than sex and anthropometric differences are believed to influence 6MWT results, including cultural norms regarding usual walking pace, mood, the motivation of the subject and/or technician, and characteristics of the provided walking track [41]. Short stature and the ambient heat and humidity may also have contributed to the low 6MWTs here. The SGRQ results were lower (better) among volunteers in this study (median 0 units, Table 3, Figure 1), compared with other healthy populations, in whom overall mean scores of 12 [43] and 9.67 [44] have been reported. The younger ages of controls in this study may explain the difference, but local expectations regarding personal health and wellbeing may also be important. Thus the establishment of locally-relevant reference ranges is especially important for both 6MWT and SGRQ, for men and women of both ethnic groups.

Compared with controls, exercise capacity was poor in TB patients, including at treatment completion. Improvement over time was only 14.7%, the same improvement as observed in %predicted FEV_1_. This simple and inexpensive test of functional capacity has been previously tested in TB [8,45], but without comparison with controls. High SGRQ among TB patients indicated substantially impaired QoL at baseline, but major improvement occurred over time. As in the controls, SGRQ scores in TB patients in this study (total score median 36.9 units) are similar to or better than previously reported in TB. We previously reported a mean total SGRQ score of 45.4 in Timika PTB patients at diagnosis [8]. A mean score of 23.5 was reported in a study of post-TB QoL in the USA [14], whereas our study participants had a median score of 4.1 at treatment completion (Figure 4B). Our TB participants experienced an overall decrement of >30 points; a major (87%) improvement. Thus despite their limitations in 6MWT and FEV_1_, and persisting symptoms, and despite the SGRQ result still not recovering to that seen in their healthy counterparts, PTB patients in this study nevertheless perceived their QoL to be relatively good by 6 months. This could be a reason for treating clinicians to under-appreciate the extent of objective residual disability if they use subjective assessments (asking patients if they feel better). In an environment such as Papua where burden of disease is high [46], reported wellness may be higher in the setting of low-grade symptoms or functional limitations, compared with people in more affluent settings with different expectations about their health. 

We did not find an association between smoking and increased risk of post-TB residual disability, for which there could be several explanations. Smoking cessation may be more common in those susceptible to the adverse effects of smoking, including those with more advanced PTB, and smoking may be more available to those of higher economic status. Indeed, a ‘healthy smoker’ effect has been noted in this environment before [8]. The young age of the majority of patients may mean low opportunity for established smoking-related lung damage to have developed, supported by the finding that airways obstruction (FEV1/FVC ratio <0.7) was uncommon. The fact that smoking rates were high (76% of males were current or ex-smokers) meant that the sample size of non-smokers for comparison was small.

A possible limitation of the study is recall bias, which could affect recollection of illness duration prior to TB treatment, or symptoms which occurred since the last appointment. However the prospective nature of the study minimises recall bias for most measures. Not all participants attended all appointments, but we restricted serial analyses to those for whom serial results were available. Hepatoxicity was not assessed due to liver function not being routinely tested in this setting; multiple other studies have previously examined rates of and risk factors for hepatotoxicity, and our intention was to focus on neglected and non-severe events, in particular, symptoms which can be assessed inexpensively. The modest number of control subjects means that the healthy reference ranges may not be completely representative of the larger population, but reassuringly, our controls showed similar pulmonary function to 107 previously-studied people in Timika, from whom predicted FEV_1_ data were obtained [13]; however, 6MWT and SGRQ were not tested in the previous study. 

We have shown that standard PTB treatment is highly successful in reducing symptoms, improving functional capacity, and enhancing quality of life, as expected. However, our study reveals the extent of the morbidity which persists at treatment completion, which would be overlooked if only routine tests (sputum and chest x-rays) were performed. Assigning a ‘successful’ treatment outcome to a patient who has been microbiologically cured ignores their functional state. Morbidity is likely to be underestimated both by treating clinicians and by patients, if objective functional measures are not performed. Patients here reported major improvements in well-being by 6 months compared to how sick they had been at diagnosis, but may have persisting impairment lifelong [3,7-11,14]. At a global level, calculations of PTB-related burden of disease should take this into account. At the individual level, people who are ‘cured’ of pulmonary TB may still have important health care requirements relating to residual lung disease; understanding residual disability could assist in planning ongoing patient care including anti-smoking, vaccination (pneumococcal and influenza) and pulmonary rehabilitation advice, where this exists. Our findings emphasise the key importance of early case detection and treatment to reduce the likelihood of residual impairment.
